# Heterofermentative Lactic Acid Bacteria Enhance the Aerobic Stability of Sweet Sorghum Silage

**DOI:** 10.1111/1751-7915.70262

**Published:** 2025-11-08

**Authors:** Muhammad Tahir, Tianwei Wang, Zhiquan Liu, Yongkai Luo, Zhihui Fu, Shanji Liu, Jin Zhong

**Affiliations:** ^1^ State Key Laboratory of Microbial Resources Institute of Microbiology, Chinese Academy of Sciences Beijing China; ^2^ School of Life Sciences University of Chinese Academy of Sciences Beijing China; ^3^ State Key Laboratory of Vegetation and Environmental Change Institute of Botany, Chinese Academy of Sciences Beijing China; ^4^ Shandong Key Laboratory of Eco‐Environmental Science for Yellow River Delta Shandong University of Aeronautics Binzhou China

**Keywords:** Feed safety, Microbial suppression, Nutrient retention, Silage preservation, Synergistic interaction

## Abstract

Sweet sorghum ( *Sorghum bicolor* L.) silage is highly prone to aerobic spoilage due to its high sugar content, leading to significant nutritional losses. This study applied absolute microbial quantification, providing novel insights into how *Lactobacillus buchneri* and *Lactobacillus hilgardii* , alone or in combination, influence microbial succession and improve the aerobic stability of sweet sorghum silage. The treatments included: (1) control (CK, sterilised water); (2) *Lactobacillus buchneri* NX205 (LB); (3) *Lactobacillus hilgardii* M1814 (LH); and (4) a combination of LB and LH (LBLH). After 60 days of ensiling, lactic acid bacteria (LAB)‐inoculated groups exhibited significantly lower pH, butyric acid and ammonia‐N (except for the LB group), along with higher acetic acid compared with the CK group (*p* < 0.05), whereas lactic acid and propionic acid contents did not differ significantly among treatments (*p* > 0.05). LAB inoculation significantly improved aerobic stability, with the LBLH group exhibiting the longest stability period compared to CK, LB and LH groups (462 h; *p* < 0.005). During aerobic exposure, the LBLH group delayed nutritional and fermentation losses by maintaining lower pH and ammonia‐N levels while sustaining higher lactic and acetic contents compared to CK, LB and LH groups. Microbial analysis showed that LBLH reshaped bacterial and fungal communities, with *Gluconobacter oxydans* prevailing among bacteria and *Zygosaccharomyces bailii* and *Penicillium paneum* dominating fungi. Functional pathway prediction further revealed enrichment in carbohydrate degradation, xenobiotic metabolism and energy utilisation in LAB‐inoculated silages. Collectively, these results demonstrate that heterofermentative LAB, particularly the LBLH combination, enhances sweet sorghum silage quality by improving aerobic stability and regulating microbial succession.

## Graphical abstract

Aerobic deterioration of sweet sorghum silage is a major constraint in the feed value chain. This study presents a proof of concept for using heterofermentative LAB to improve the aerobic stability of sweet sorghum silage. Combined inoculation with *Lactobacillus buchneri* NX205 and *Lactobacillus hilgardii* M1814 not only ensures optimal ensiling conditions for sweet sorghum but also enhances its silage resistance to spoilage upon aerobic exposure.

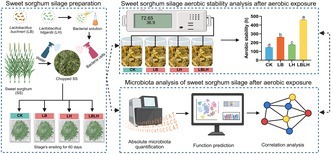

## 1 Introduction

Sweet sorghum ( *Sorghum bicolor* L.), a resilient C4 crop with high biomass and sugar‐rich stems, is distinguished by its remarkable tolerance to drought and saline‐alkaline soils (Xu et al. 2024). Its dual‐purpose grain and stem harvests support diverse uses – food, feed, biofuel, liquor and forage – enabling integrated industrial pipelines for bio‐ethanol, farming and chemicals (Kumar et al. 2024; Mukondwa et al. 2025). Since the early 20th century, the US, Brazil, Australia, India and China have advanced sweet sorghum breeding, cultivation and processing, boosting its bio‐industrial and feeding potential (Zhang et al. 2024). The forage derived from sweet sorghum can be preserved as silage or hay, with silage being the preferred option due to its fermentation benefits. Unlike hay, which depends mainly on dehydration, silage preservation involves a complex fermentation process influenced by a range of environmental factors such as moisture content, temperature and microbial activity (Haselmann et al. 2020). Although sweet sorghum silage naturally ferments well due to its high water‐soluble carbohydrate (WSC) content, its aerobic stability declines during aerobic exposure as yeasts rapidly deplete these sugars. Hence, there is a need to provide a strategy to reduce the aerobic deterioration and improve the aerobic stability of sweet sorghum.

Aerobic spoilage in silage occurs when oxygen exposure promotes the proliferation of aerobic microorganisms, such as filamentous fungi and yeasts (Shuo et al. 2024). These microbes degrade lactic acid (LA), a key fermentation product, compromising silage quality and nutritional value (Bernardes et al. 2021). Sweet sorghum silage is prone to rapid spoilage upon aerobic exposure due to microbial activity and oxygen‐driven biochemical changes. To mitigate these issues, lactic acid bacteria (LAB) serve as effective natural preservatives in livestock feed by producing antifungal metabolites (Crowley et al. 2013; Sharma et al. 2022). Heterofermentative LAB, in particular, improve aerobic stability by converting LA into acetic acid (AA), which further lowers pH and suppresses yeast and mould growth (Arriola, Oliveira, et al. 2021; Tahir et al. 2025; Wu et al. 2023). Although yeasts tolerate a wide pH range (optimally 4.0–6.0), heterofermentative LAB depress silage pH below 4.0 through AA production (Reis et al. 2018). This pH reduction, coupled with AA's antimicrobial activity, synergistically inhibits yeast and mould proliferation (Pang et al. 2024). In corn silage, inoculation with *Lactobacillus buchneri* increased 1,2‐propanediol and AA concentrations after 90 days of fermentation, thereby enhancing aerobic stability (Huang et al. 2021). Similarly, inoculating sorghum forage with *Lactobacillus buchneri* or *Lactobacillus hilgardii* elevated AA and 1,2‐propanediol, reduced lactate levels and the lactate‐to‐acetate ratio, and improved aerobic stability (Arriola, Vyas, et al. 2021). Moreover, co‐inoculation with obligate heterofermentative strains of *Lactobacillus buchneri* or *Lactobacillus hilgardii* has been shown to further enhance aerobic stability in corn silage under laboratory conditions (da Silva et al. 2021; Drouin et al. 2019). Despite these documented benefits (Blajman et al. 2018; Ferrero et al. 2021; Yin et al. 2023, 2024), the efficacy of such inoculants in sweet sorghum silage – characterised by distinct sugar and fibre dynamics – remains underexplored.

The ensiling process is driven by microbial metabolisms, which play pivotal roles in enhancing silage fermentation quality and nutritional value through substrate transformation (Ke et al. 2025). Characterising the quantitative role of microbial communities in silage through absolute abundance metrics is crucial for achieving accurate measurements of individual microbial taxa (Wang et al. 2021). Unlike relative abundance, which may obscure key microbial dynamics due to fluctuations in total community size, absolute abundance offers a definitive measure of each organism's numerical dominance and associated metabolic activity (Barlow et al. 2020). This paradigm shift from relative to absolute quantification not only enhances our understanding of microbial community structure but also enables targeted strategies to optimise silage fermentation.

In this study, we applied heterofermentative LAB strains – *Lactobacillus buchneri* NX205 and *Lactobacillus hilgardii* M1814 – to ensile sweet sorghum silage. We hypothesised that these heterofermentative LAB would improve the aerobic stability of the silage by increasing AA production, thereby reducing aerobic spoilage. Our objective was to assess the effects of heterofermentative LAB on fermentation quality, nutritional characteristics and aerobic stability using absolute quantification microbiome analysis, which is crucial for improving the utilisation of sweet sorghum silage by livestock.

## 2 Materials and Methods

### 2.1 Raw Material and Lactic Acid Bacteria Inoculants

Sweet sorghum (Ketian 14; hard dough stage) was harvested from the experimental field of the Yellow River Delta Modern Agricultural Technology Innovation Center, Dongying, Shandong Province, China on October 27, 2023. The cultivation site experiences a warm‐temperate continental monsoon climate characterised by distinct seasons, with 70% of the annual 560 mm precipitation occurring between July and September. Mean temperatures range from −2.8°C in January to 26.7°C in July (annual average: 12.9°C). During the dry season (April–June), high evaporation rates drive upward movement of water and soluble salts from shallow groundwater to the root zone, resulting in elevated surface soil moisture and salinity (Xu and Li 2025). The coastal wetlands of the Yellow River Delta feature predominantly sandy clay loam soils that transition gradually from fluvo‐aquic to saline types (Han et al. 2018). The harvested fresh forage material was processed into 1–2 cm particles using a forage chopper. The pH value and chemical characteristics of sweet sorghum prior to ensiling were as follows (Table 1): pH, 5.24; dry matter (DM), 33.08%; crude protein (CP), 5.13% DM; WSC, 24.47% DM; neutral detergent fibre (NDF), 49.93% DM; and acid detergent fibre (ADF), 29.96% DM. The LAB, aerobic bacteria and yeast counts were 8.91, 7.41 and 8.96 log_10_ CFU/g fresh matter (FM), respectively. The *Lactobacillus buchneri* NX205 [currently known as *Lentilactobacillus buchneri* NX205 (LB)] and *Lactobacillus hilgardii* M1814 [currently known as *Lentilactobacillus hilgardii* M1814 (LH)] were used as LAB inoculants. Both strains were initially cultured on de Man, Rogosa and Sharpe (MRS) agar at 37°C for 48 h under anaerobic conditions (Tahir et al. 2025). A single colony was inoculated into 5 mL of MRS broth and incubated anaerobically at 37°C until reaching an optical density (OD_600_) of 1.0. Following this, the bacterial culture was transferred to a 500 mL flask for further growth. Finally, a tenfold dilution plating method was employed to determine the viable bacterial count.

**TABLE 1:** Characteristics of fresh sweet sorghum prior to ensiling.

Items	Fresh sweet sorghum
pH and chemical composition	
pH value	5.24
Dry matter (%)	33.08
Crude protein (%DM)	5.13
Water‐soluble carbohydrates (%DM)	24.47
Neutral detergent fibre (%DM)	49.93
Acid detergent fibre (%DM)	29.96
Microbial counts	
Lactic acid bacteria (log_10_ CFU/g FM)	8.91
Aerobic bacteria (log_10_ CFU/g FM)	7.48
Yeasts (log_10_ CFU/g FM)	8.94

*Note:* Data is the mean of three replicates.

### 2.2 Silage Preparation

Before initiating the silage experiment, we cleaned the experimental area with 75% ethanol to eliminate potential contaminants that could interfere with the fermentation process of silage. The chopped sweet sorghum material was treated as follows: (1) CK (control), (2) LB, (3) LH and (4) LBLH (a combination of LB and LH). The LAB inoculants were applied at a concentration of 10^6^ CFU/g fresh weight (FW) by spraying onto the chopped sweet sorghum, while the CK group received an equal amount of sterilised water. Approximately 500 g of treated sweet sorghum material (12 replicates per group) was packed into polyethylene bags (30 × 40 cm) and vacuum‐sealed. All silage samples were kept at room temperature, allowing for 60 days of ensiling. After ensiling, three replicates per group were opened to assess the chemical characteristics, fermentation quality and culture‐based microbial populations. The remaining nine replicates of each group (*n* = 9) were pooled into three composite samples (1.5 kg each) to ensure sufficient material for aerobic stability testing while maintaining biological relevance. Silage quality data and sequencing data after aerobic exposure were generated from composite samples (*n* = 3), and each composite was treated as an independent unit (*n* = 3) in statistical analyses.

### 2.3 Chemical Composition and Fermentation Quality Analyses

Fresh and ensiled silage samples underwent drying in a forced‐air oven at 65°C for 72 h to determine DM content. After drying, all fresh and silage samples were ground to fine powder for subsequent analysis. The CP, NDF and ADF contents were assessed following the procedures outlined by the Association of Official Analytical Chemists (Van Soest et al. 1991). The WSC content was measured using an automated colorimetric method (Arthur Thomas 1977). To assess fermentation quality, 10 g of each silage sample was homogenised with 90 mL of sterilised water and shaken for 30 min (Wang et al. 2022). The pH of the resulting solution was immediately recorded using a pH metre (model: LEICI pH S‐3 C, Shanghai Yitian Scientific Instrument Co. Ltd., Shanghai, China). Organic acids, including LA, AA, propionic acid (PA) and butyric acid (BA), were quantitatively assessed using high‐performance liquid chromatography (HPLC) (model 1200; Agilent, California, USA). The mobile phase was maintained at 55°C with a flow rate of 0.6 mL/min, using a 0.005 M sulfuric acid (H_2_SO_4_) solution. Ammonia nitrogen (ammonia‐N) content was assessed using the ninhydrin colorimetric and phenol‐hypochlorite methods (Broderick and Kang 1980).

### 2.4 Cultured‐Based Microbial Population Analysis

The culture‐based microbial population was carried out according to a previously reported method (Tahir et al. 2023). A portion of the silage extract prepared for fermentation quality indicators analyses was filtered through a single layer of sterilised gauze to remove large particulates, and then serial dilutions were prepared for microbial counts. To quantify specific microbial groups, the filtrate was spread onto selective media: MRS for LAB, Luria–Bertani (LB) agar for aerobic bacteria and potato dextrose agar (PDA) for yeasts. MRS and LB plates were maintained anaerobically at 37°C for 48 h, while PDA plates were incubated aerobically at 30°C for 72 h. Microbial counts were recorded as colony‐forming units per gram of fresh material (CFU/g FM) and subsequently log‐transformed to assist the statistical analysis.

### 2.5 Aerobic Stability Analysis

The aerobic stability analysis was carried out after 60 days of ensiling according to a previously reported method (Wang et al. 2020). All nine silage samples were opened after 60 days of ensiling and then bulked into three composite samples for each group, with each composite weighing approximately 1.5 kg. The silage materials of 1.5 kg were loaded into 3‐L sterilised heat‐insulating containers without compaction and kept at room temperature. A multi‐channel temperature recorder (model: MDL‐1048 A; Shanghai Tianhe Automation Instrument Co. Ltd., Shanghai, China) was used to record room temperature and temperature within the containers for a consecutive 22 days. To minimise contamination and moisture loss, we covered each container with two layers of cheesecloth. We defined the aerobic stability as the time until the temperature within the silage rises more than 2°C above ambient temperature (Liu et al. 2025). Sub‐samples from each replicate were collected after 2, 5, 8 and 22 days of aerobic exposure to evaluate the changes in nutritional characteristics, fermentation quality and cultivable microbes, while microbial diversity assessments were performed only on days 8 and 22.

### 2.6 Absolute 16S and ITS Sequencing

The microbial diversity of sweet sorghum silage was evaluated after 8 and 22 days of aerobic exposure. For microbial DNA extraction, 10 g of aerobic silage sample was homogenised with 40 mL of sterile PBS (4°C) using orbital shaking (120 rpm, 30 min). The homogenate was sequentially filtered through four‐layer sterile gauze to remove particulate matter, followed by centrifugation (12,000 rpm, 5 min, 4°C) to pellet microbial cells. The resulting bacterial pellet was then processed for total genomic DNA extraction using a commercial soil DNA isolation kit (Yeasen, 18815ES50, Shanghai, China) according to the manufacturer's protocol. The integrity of DNA was verified by agarose gel electrophoresis. Quantification and purity of DNA were determined using a Nanodrop 2000 spectrophotometer and Qubit 3.0 Fluorometer, respectively. Paired‐end sequencing (2 × 250 bp) was conducted on the Illumina MiSeq platform (Genesky Biotechnologies Inc., Shanghai) following standard protocols. For bacterial community profiling, the V3‐V4 regions of the 16S rRNA gene were amplified with primers 341F (CCTACGGGNGGCWGCAG) and 805R (GACTACHVGGGTATCTAATCC). Fungal communities were characterised by sequencing the ITS region with the primers ITS1F (5′–CTTGGTCATTTAGAGGAAGTAA–3′) and ITS2R (5′–GCTGCGTTCTTCATCGATGC–3′). The raw read sequences were processed in QIIME2 (Bolyen et al. 2019). The adaptor and primer sequences were trimmed using the cutadapt plugin. DADA2 plugin was used for quality control and to identify amplicon sequence variants (ASVs) (Callahan et al. 2016). Taxonomic assignments of ASV representative sequences were performed with a confidence threshold of 0.8 by a pre‐trained Naive Bayes classifier, which was trained on the Greengenes (version 13.8). Then the spike‐in sequences were identified and reads were counted. A standard curve for each sample was generated based on the read‐counts versus spike‐in copy number, and the absolute copy number of each ASV in each sample was calculated by using the read‐counts of the corresponding ASV. Since the spike‐in sequence is not a component of the sample microbiota, the spike‐in sequence needed to be removed in the subsequent analysis (Jiang et al. 2019). Bacterial functional potential was predicted via PICRUSt2, with Kyoto Encyclopedia of Genes and Genomes (KEGG) pathways visualised in a heatmap (generated using Chiplot; https://www.chiplot.online/). Raw sequences are accessible in the National Center for Biotechnology Information (NCBI) Sequence Read Archives (bacteria: PRJNA1266375; fungi: PRJNA1266384).

### 2.7 Statistical Analysis

The experimental data were processed using Excel 2019 and analysed with SPSS software. Chemical composition, fermentation quality and cultivable microbial counts data after 60 days of ensiling were assessed using one‐way analysis of variance, whereas data from aerobic exposure were analysed via two‐way analysis of variance. Post hoc comparisons of means were conducted with Duncan's test (*p* < 0.05) to identify significant differences. Graphical representations of the results were generated using GraphPad Prism 8.0 for enhanced data visualisation.

## 3 Results

### 3.1 Physicochemical Profiles and Microbial Counts of Sweet Sorghum Silage in Response to Heterofermentative LAB Additives

**FIGURE 1:**
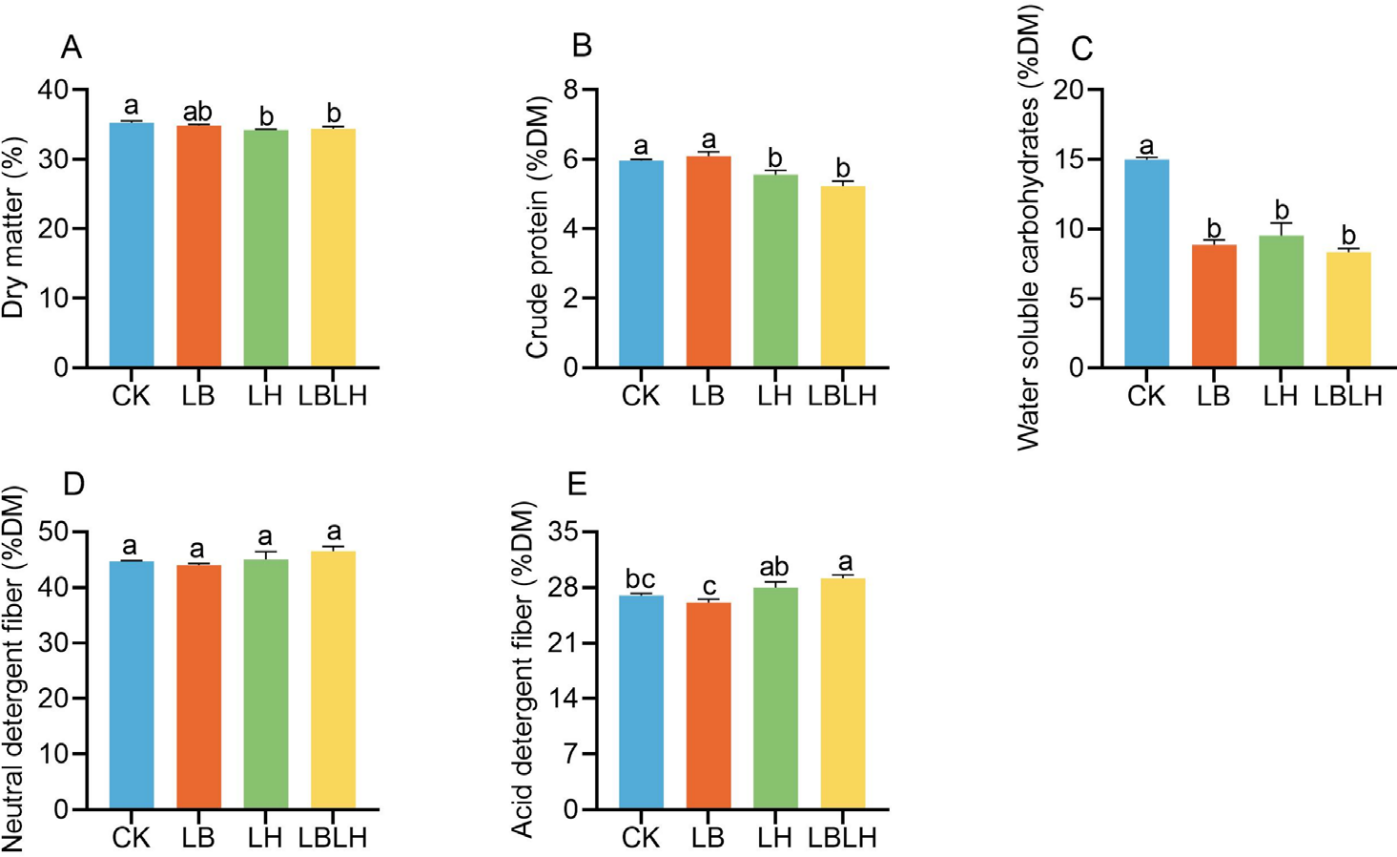
Chemical composition of sweet sorghum silage after 60 days of ensiling in response to heterofermentative lactic acid bacteria additive. (A) dry matter, (B) crude protein, (C) water‐soluble carbohydrates, (D) neutral detergent fibre, (E) acid detergent fibre. CK, sterilised water; LB, *Lactobacillus buchneri* NX205; LH, *Lactobacillus hilgardii* M1814; LBLH, combination of LB and LH. Data is the mean of three replicates. The small letters indicate the statistical difference among treatments which was employed at 0.05 probability level.

**FIGURE 2:**
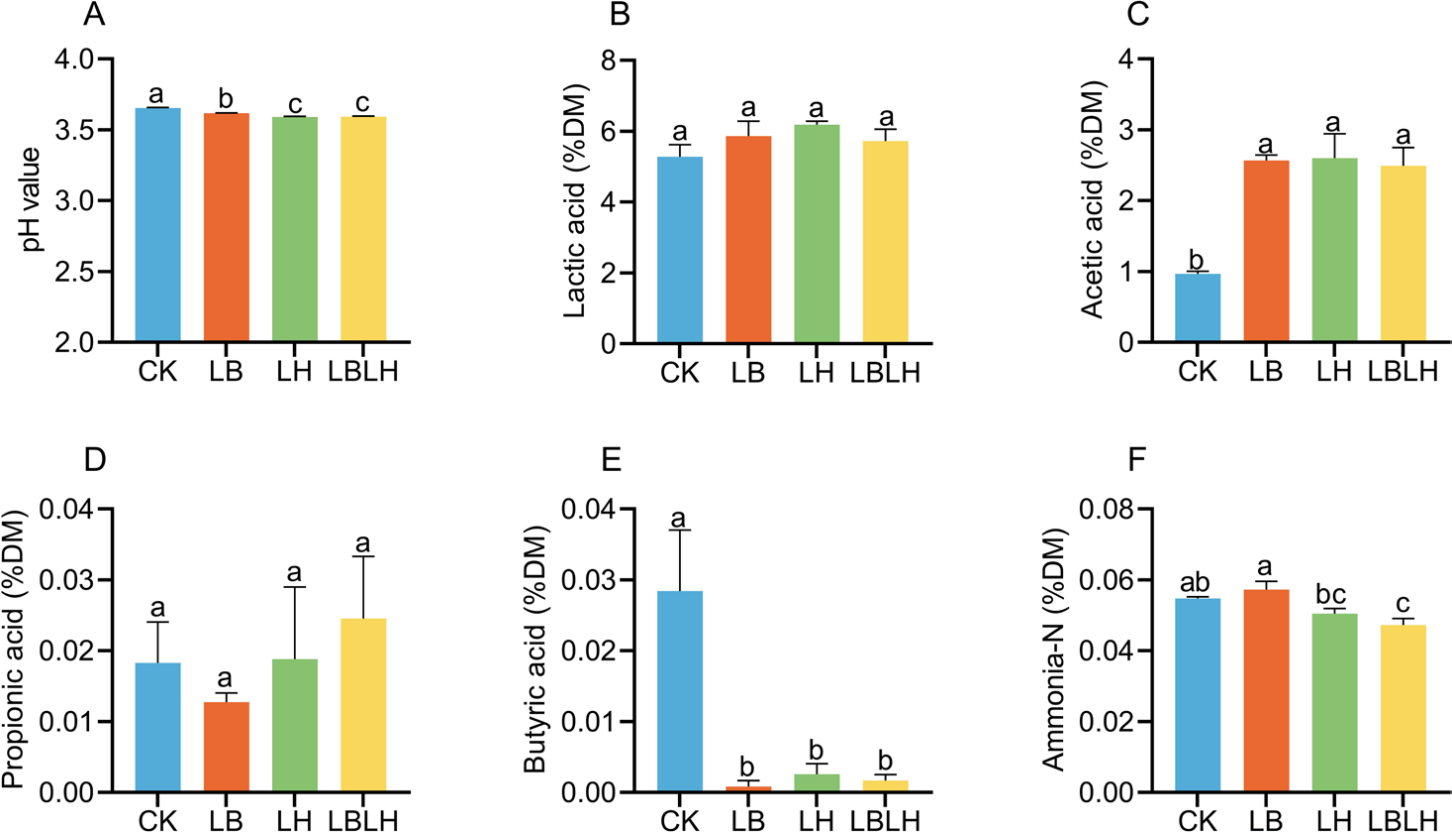
Fermentation quality of sweet sorghum silage after 60 days of ensiling in response to heterofermentative lactic acid bacteria additive. (A) pH value, (B) lactic acid, (C) acetic acid, (D) propionic acid, (E) butyric acid, (F) ammonia‐N. CK, sterilised water; LB, *Lactobacillus buchneri* NX205; LH, *Lactobacillus hilgardii* M1814; LBLH, combination of LB and LH. Data is the mean of three replicates. The small letters indicate the statistical difference among treatments which was employed at 0.05 probability level.

The physicochemical profiles of sweet sorghum silage treated with heterofermentative LAB after 60 days of ensiling are shown in Figures 1 and 2, whereas the corresponding cultivable microbial populations are presented in Figure S1. Compared to the CK group, the LH and LBLH groups had significantly lower DM and CP contents (*p* < 0.05), but the LB group showed no significant difference (Figure 1A,B). All LAB‐inoculated groups showed significantly lower WSC contents compared to the CK group (*p* < 0.05; Figure 1C). Notably, the LB group resulted in a significantly lower ADF content compared to the CK, LH and LBLH groups (*p* < 0.05; Figure 1E), whereas NDF content remained unaffected by the groups (Figure 1D). All LAB‐inoculated groups demonstrated significantly higher AA and lower BA contents compared to the CK group (*p* < 0.05; Figure 2C,E), while LA and PA contents did not differ significantly among the groups (*p* > 0.05; Figure 2B,D). As a result, all LAB‐inoculated groups exhibited lower pH values than the CK group (*p* < 0.05; Figure 2A), with the LH and LBLH groups demonstrating significantly reduced ammonia‐N content relative to both the CK and LB groups (*p* < 005; Figure 2F). Additionally, all LAB‐inoculated groups exhibited lower aerobic bacteria counts (*p* < 0.05; Figure S1B), with the LB group exhibiting significantly lower yeast counts than the CK, LH and LBLH groups (*p* < 0.05; Figure S1C), whereas LAB counts showed no significant difference among the groups (Figure S1A).

### 3.2 Aerobic Stability of Sweet Sorghum Silage in Response to Heterofermentative LAB Additives

**FIGURE 3:**
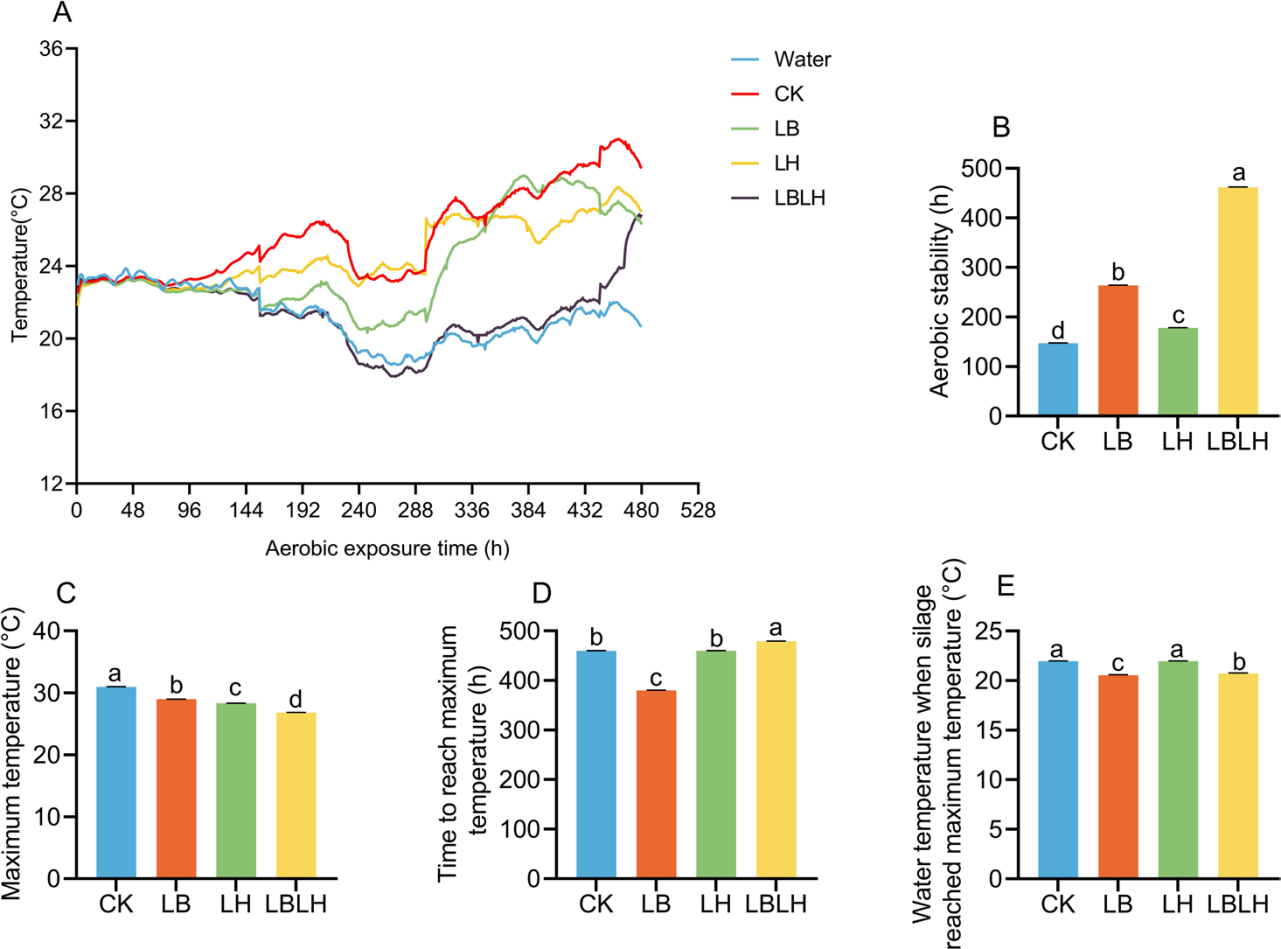
Aerobic stability of sweet sorghum silage in response to heterofermentative lactic acid bacteria additive. (A) temperature curve during aerobic exposure, (B) aerobic stability, (C) maximum temperature, (D) time to reach maximum temperature, (E) water temperature when silage reached maximum temperature. CK, sterilised water; LB, *Lactobacillus buchneri* NX205; LH, *Lactobacillus hilgardii* M1814; LBLH, combination of LB and LH. Data is the mean of three replicates. The small letters indicate the statistical difference among treatments which was employed at 0.05 probability level.

The aerobic stability and change in temperature during aerobic exposure of sweet sorghum silage in response to heterofermentative LAB are presented in Figure 3. The CK group began to deteriorate after 147 h, while the LB and LH groups showed delayed deterioration, starting at 264 h and 178 h, respectively (Figure 3A). Notably, the LBLH group exhibited significantly higher aerobic stability of 462 h compared to the other groups (*p* < 0.05; Figure 3B). The CK group showed maximum temperature caused by secondary fermentation compared to LAB‐inoculated groups, with the LBLH group exhibiting significantly lower maximum temperature compared to other groups (*p* < 0.005; Figure 3C). Additionally, the LBLH group took significantly longer to reach its maximum temperature (479 h) than the other groups (*p* < 0.05; Figure 3D). The water temperature of CK and LH groups was significantly higher compared to LB and LBLH groups when aerobic silages reached maximum temperature (*p* < 0.05; Figure 3E).

### 3.3 Physicochemical Profiles and Microbial Counts of Sweet Sorghum Silage During Aerobic Exposure in Response to Heterofermentative LAB Additives

**FIGURE 4:**
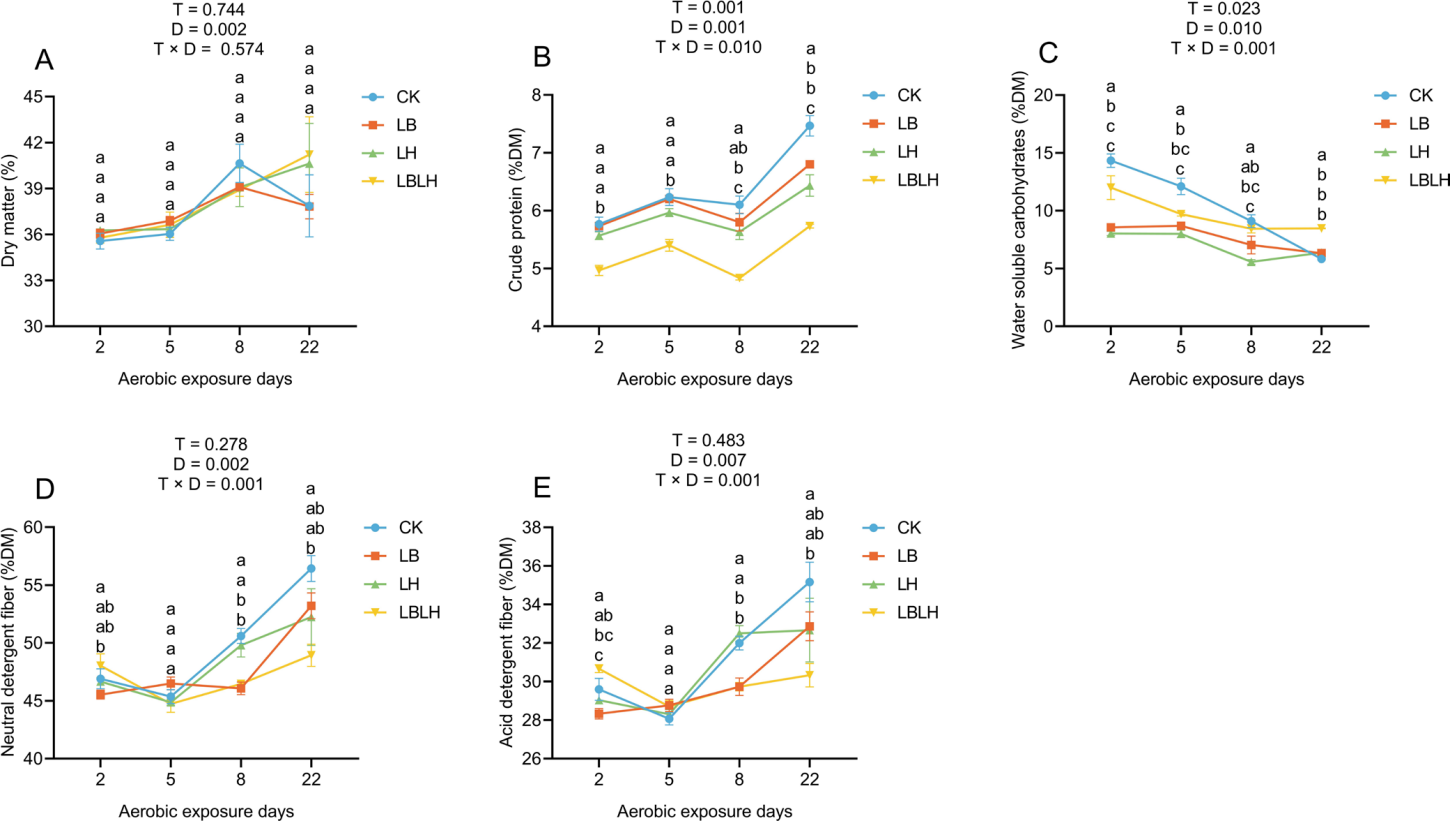
Chemical composition of sweet sorghum silage after aerobic exposure in response to heterofermentative lactic acid bacteria additive. (A) dry matter, (B) crude protein, (C) water‐soluble carbohydrates, (D) neutral detergent fibre, (E) acid detergent fibre. CK, sterilised water; LB, *Lactobacillus buchneri* NX205; LH, *Lactobacillus hilgardii* M1814; LBLH, combination of LB and LH; T, treatment; D, aerobic exposure days; T × D; interactive effect between treatments and aerobic exposure days. Data is the mean of three replicates. The small letters indicate the statistical difference among treatments at same time point, which was employed at 0.05 probability level.

**FIGURE 5:**
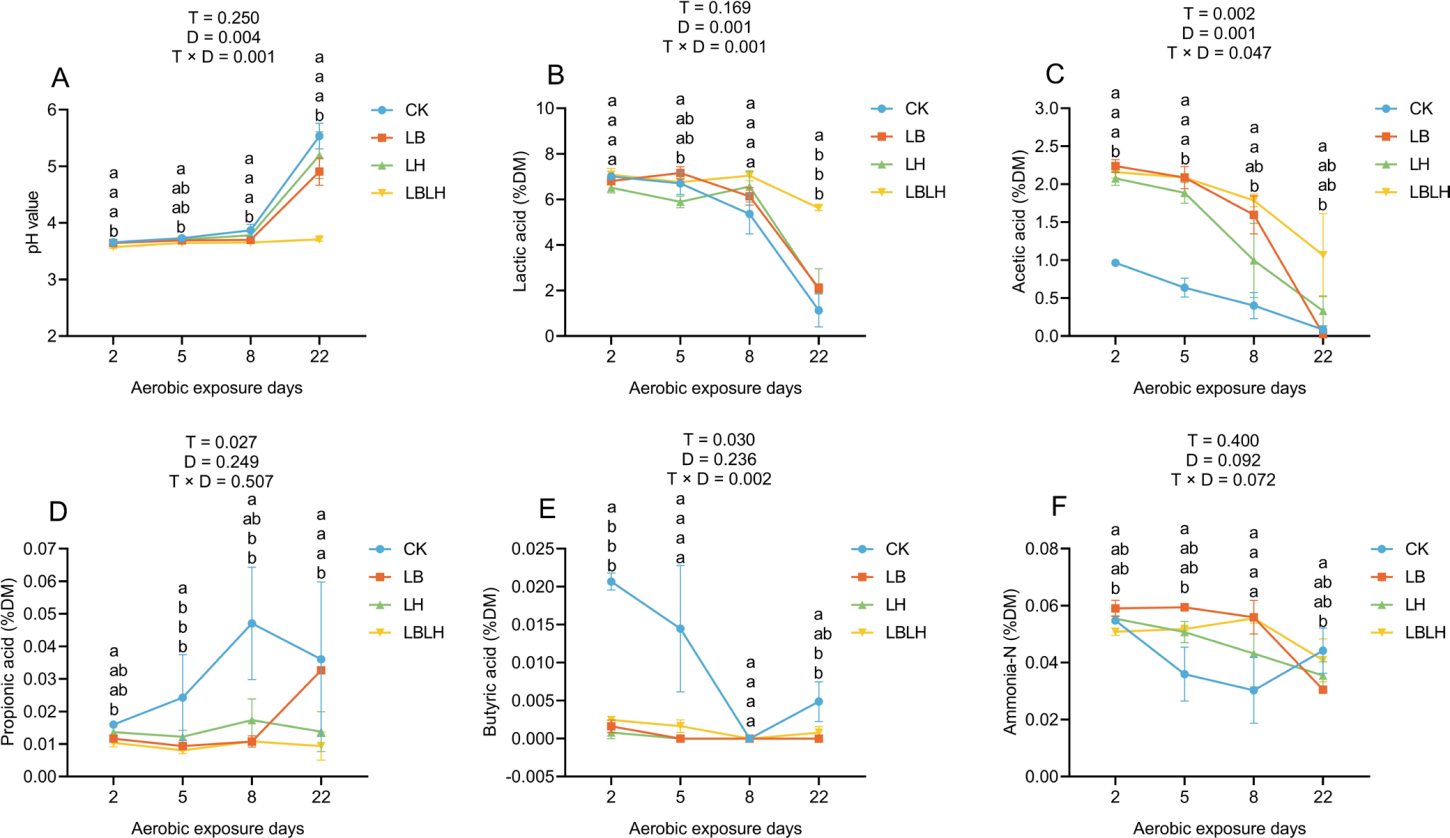
Fermentation quality of sweet sorghum silage after aerobic exposure in response to heterofermentative lactic acid bacteria additive. (A) pH value, (B) lactic acid, (C) acetic acid, (D) propionic acid, (E) butyric acid, (F) ammonia‐N. CK, sterilised water; LB, *Lactobacillus buchneri* NX205; LH, *Lactobacillus hilgardii* M1814; LBLH, combination of LB and LH; T, treatment; D, aerobic exposure days; T × D; interactive effect between treatments and aerobic exposure days. Data is the mean of three replicates. The small letters indicate the statistical difference among treatments at same time point, which was employed at 0.05 probability level.

The physicochemical profiles of sweet sorghum silage treated with heterofermentative LAB after aerobic exposure are shown in Figures 4 and 5, whereas the corresponding cultivable microbial populations are presented in Figure S2. DM content increased with aerobic exposure time, but no T × D interaction was detected, although a decline was observed in the CK and LB groups after 8 days of aerobic exposure (Figure 4A). CP content displayed a significant T × D interaction (*p* = 0.010), as the CK group retained significantly higher CP content than LAB‐inoculated groups after 22 days of aerobic exposure (*p* < 0.05; Figure 4B), suggesting that heterofermentative LAB were less effective in conserving protein under extended oxygen stress. WSC content also showed a strong T × D interaction (*p* = 0.001): the CK group maintained higher WSC initially but underwent sharp declines, whereas LAB‐inoculated silages exhibited consistently lower WSC (except for LBLH at 22 days of aerobic exposure), reflecting more complete utilisation of sugars during fermentation (Figure 4C). Both the NDF and ADF contents displayed significant T × D interactions (*p* = 0.001), and their contents increased significantly in the CK group but remained more stable in LAB‐inoculated groups, especially in LBLH (Figure 4D,E), indicating that LAB inoculation mitigated the relative enrichment of structural fibre that typically accompanies the loss of soluble nutrients during spoilage.

The pH value displayed a significant T × D interaction (*p* = 0.001), remaining stable until day 8 before rising sharply, with the highest value observed in the CK group, whereas LAB‐inoculated groups, particularly LBLH, maintained significantly lower pH (*p* < 0.05; Figure 5A), reflecting enhanced resistance to aerobic spoilage. The LA and AA contents showed significant T × D interactions (for LA, *p* = 0.001; for AA, *p* = 0.047; Figure 5B,C). The LA contents declined across all groups, but its depletion was most pronounced in CK, LB and LH groups, while the LBLH group preserved significantly higher content until day 22 (*p* < 0.05; Figure 5B), suggesting that combined LB and LH inoculation delayed the loss of antifungal protection. The AA content followed a similar trend, with the LBLH group maintaining significantly higher content than the CK group (*p* < 0.05; Figure 5C). In contrast, PA accumulated sharply in the CK group but remained low and stable in LAB‐inoculated groups (*p* = 0.027; Figure 5D). Likewise, BA content declined in LAB‐inoculated groups but increased transiently in the CK group and T × D interaction was significant (*p* = 0.002; Figure 5E), highlighting the ability of LAB to inhibit clostridial metabolism and proteolysis under aerobic conditions. No significant T × D interaction was detected for ammonia‐N content; however, at 22 days of aerobic exposure, the LBLH group exhibited significantly lower ammonia‐N levels than the CK group (*p* < 0.05), although the difference was not significant when compared with the LB and LH groups (Figure 5F). T × D interaction was significant (*p* = 0.001) for LAB and aerobic bacteria counts, but was non‐significant (*p* = 0.623) for yeast counts (Figure S2). LAB counts declined progressively in all groups, but the decline was more pronounced in the CK group than in LAB‐treated groups, especially in the LBLH group (Figure S2A). The CK group showed a sharp increase in aerobic bacteria counts from day 5 onward, reaching the highest levels at day 22, whereas LAB‐inoculated groups, especially LBLH, showed restrained proliferation (Figure S2B), indicating that additives effectively suppressed aerobic competitors and delayed microbial‐driven deterioration. Yeast counts increased over time across all groups, but the LBLH group consistently exhibited lower counts than the other groups (*p* < 0.05; Figure S2C). The lack of interaction implies that inoculants reduced yeast growth to a similar extent throughout exposure, thereby prolonging aerobic stability.

### 3.4 Microbial Community of Sweet Sorghum Silage During Aerobic Exposure in Response to Heterofermentative LAB Additives

**FIGURE 6:**
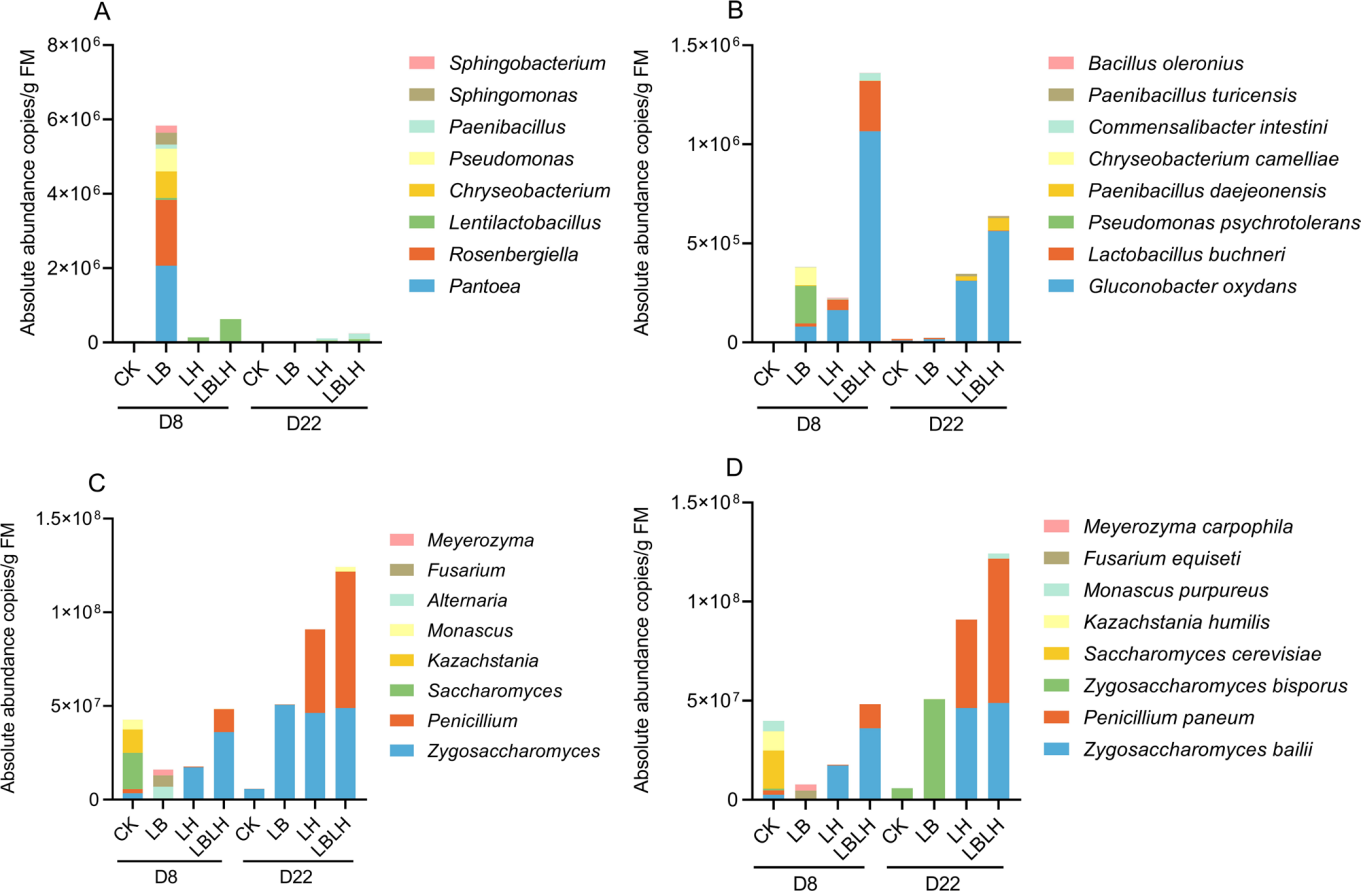
Absolute microbial community of sweet sorghum silage after aerobic exposure in response to heterofermentative lactic acid bacteria additive. (A) bacterial community at genus level, (B) bacterial community at specie level, (C) fungal community at genus level, (D) fungal community at specie level. CK, sterilised water; LB, *Lactobacillus buchneri* NX205; LH, *Lactobacillus hilgardii* M1814; LBLH, combination of LB and LH; D, aerobic exposure days.

To explore how heterofermentative LAB additives enhance the aerobic stability of sweet sorghum silage, absolute quantification 16S sequencing (AQ‐16S‐seq) was used to analyse microbial community dynamics after 8 and 22 days of aerobic exposure. The T × D interaction was not significant for bacterial alpha diversity indices (Table S1). The genera *Pantoea* and *Rosenbergiella* dominated in the LB group, whereas *Lentilactobacillus* was abundant in the LH and LBLH groups (Figure 6A). *Pseudomonas psychrotolerans* prevailed in the LB, while *Gluconobacter oxydans* and *Lactobacillus buchneri* were dominant species in the LH and LBLH (Figure 6B). The LBLH group had the highest total bacterial abundance at species level across both aerobic time points, whereas CK showed significantly lower bacterial abundance at both genus and species levels compared to other groups.

T × D interaction was significant for fungal alpha diversity indices of observed species (*p* = 0.004), Chao1 (*p* = 0.005) and ACE (*p* = 0.004) (Table S2). Compared to the CK group, the observed species, Chao1 and ACE indices decreased in LAB‐inoculated groups with prolonged aerobic exposure, suggesting that heterofermentative LAB restricted the proliferation of diverse fungal taxa. At the genus level, *Saccharomyces* and *Kazachstania* dominated the CK group, whereas *Zygosaccharomyces* and *Penicillium* were most abundant in LAB‐inoculated groups (Figure 6C). The total absolute abundance of *Zygosaccharomyces* and *Penicillium* in LAB‐inoculated groups increased substantially with prolonged aerobic exposure, peaking in the LBLH group at 22 days. In the CK group, *Saccharomyces cerevisiae* and *Kazachstania humilis* were the most prevalent species at 8 days of aerobic exposure, but were later replaced by *Zygosaccharomyces bisporus* at 22 days (Figure 6D). In LAB‐inoculated groups, *Zygosaccharomyces balii* and *Fusarium equiseti* dominated at 8 days, while *Zygosaccharomyces bisporus* became dominant in the LB group at 22 days. In contrast, *Zygosaccharomyces balii* and *Penicillium paneum* became the most abundant species in the LH and LBLH groups after 22 days. The absolute abundance of fungal species in LAB‐inoculated groups increased significantly over time, with the LBLH group exhibiting the highest abundance compared to other groups.

### 3.5 Predicted Pathways of Bacterial Communities of Sweet Sorghum Silage During Aerobic Exposure

**FIGURE 7:**
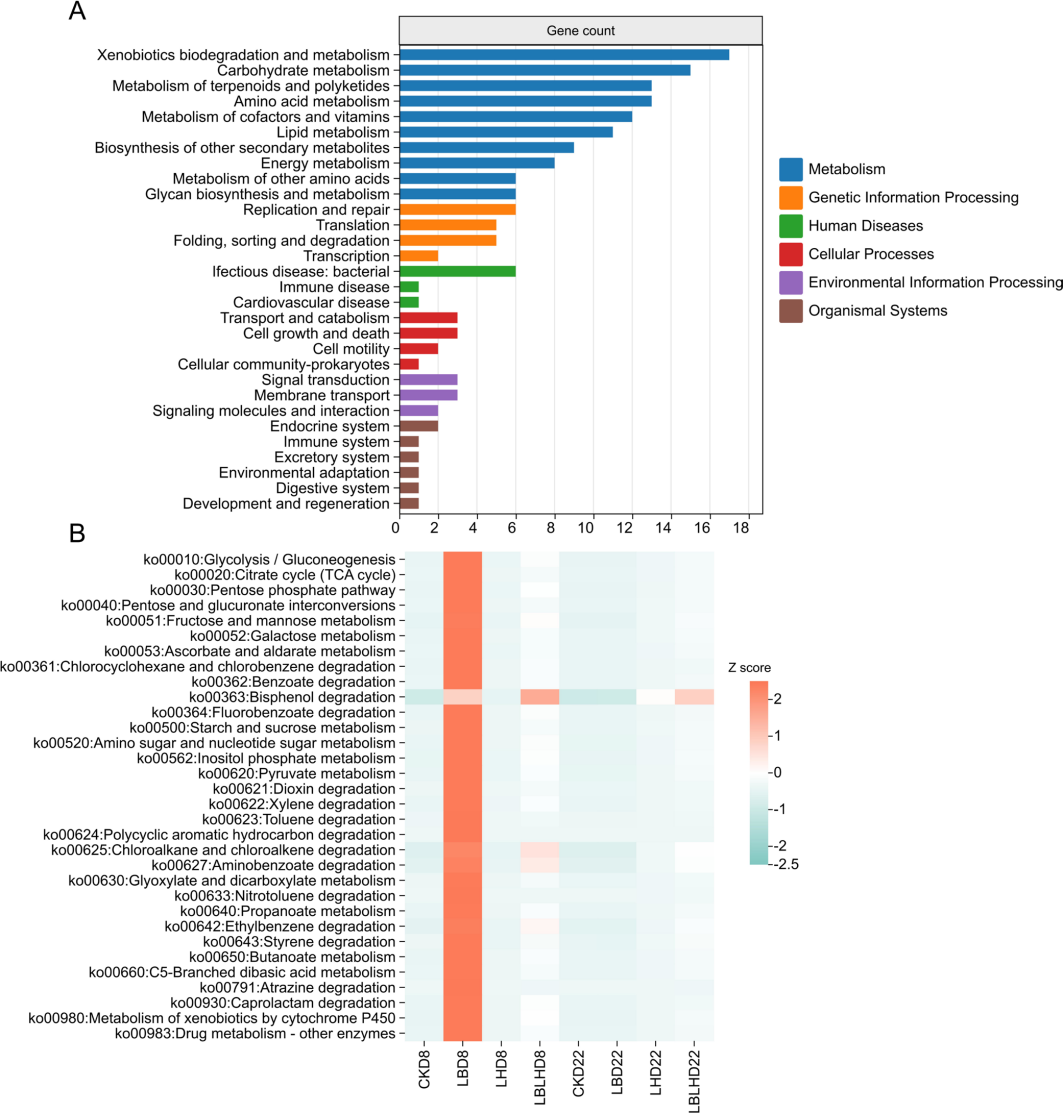
Absolute quantification 16S rRNA gene‐predicted functional profiles of sweet sorghum silage after aerobic exposure in response to heterofermentative lactic acid bacteria additive. (A) metabolic pathway at level 1 and 2, (B) metabolic pathways at level 3. CK, sterilised water; LB, *Lactobacillus buchneri* NX205; LH, *Lactobacillus hilgardii* M1814; LBLH, combination of LB and LH; D, aerobic exposure days. Legend with red and green colours represent the relative enrichment or depletion of each pathway based on *Z* score.

To gain deeper insights into the biochemical pathways employed by bacterial communities following aerobic exposure, we reconstructed functional pathways using the KEGG database. Our analysis revealed that the predominant predicted microbiome functions were closely linked to metabolism, particularly xenobiotic biodegradation and metabolism, carbohydrate metabolism, terpenoid and polyketide metabolism and amino acid metabolism (Figure 7A). A heatmap of KEGG level 3 pathways further illustrated changes in xenobiotic biodegradation and metabolism, as well as carbohydrate metabolism (Figure 7B). All pathways in the CK and LH groups were down‐regulated after aerobic exposure. In contrast, the LB group exhibited up‐regulation of nearly all pathways at 8 days of aerobic exposure, except for Ko00363 (Bisphenol degradation), which was uniquely enriched in the LBLH group.

### 3.6 Identification of the Relationships Between Microbial Community and Silage Metabolites

**FIGURE 8:**
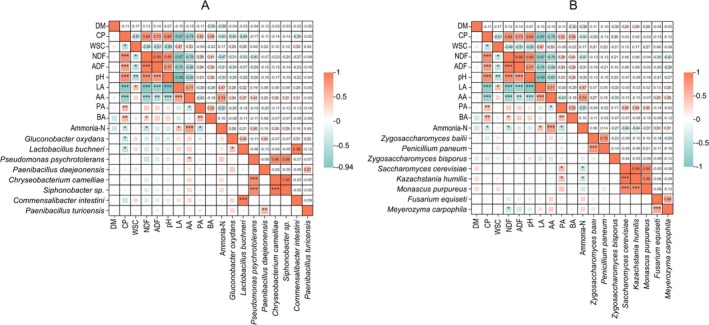
Identification of relationships between microbial community and silage quality. (A) correlation between bacterial community and silage quality (B) correlation between fungal community and silage quality. Red, positive correlation; blue, negative correlation. The square chart represents the strength of correlation coefficients, whereas the shaded colour denotes the statistical significance of these coefficients.

To further investigate the influence of microbial species on the fermentation profile and nutritional characteristics during aerobic exposure, we conducted Pearson correlation analysis between distinct microbial species and key parameters (Figure 8). AA exhibited a significant positive correlation with the bacterial species of *Pseudomonas psychrotolerans* , whereas CP showed a significant negative association with *Lactobacillus buchneri* and *Gluconobacter oxydans* (Figure 8A). PA was positively correlated with *Kazachstania humilis* and *Saccharomyces cerevisiae* , whereas NDF was negatively correlated with *Meyerozyma carpophila* (Figure 8B). Overall, *Pseudomonas psychrotolerans* , *Lactobacillus buchneri* , *Gluconobacter oxydans* , *Kazachstania humilis* and *Saccharomyces cerevisiae* may be key microbial species affecting the fermentation quality and nutritional characteristics of sweet sorghum silage during aerobic exposure.

## 4 Discussion

The primary objective of sweet sorghum silage fermentation is to produce high‐quality silage that retains nutritive value while optimising fermentation characteristics. Recently, heterofermentative LAB additives have gained attention for retaining nutritional value and fermentation quality of silages, especially after aerobic exposure (Okoye et al. 2023). This study presents a significant advancement in enhancing the aerobic stability of sweet sorghum silage by integrating quantitative microbiome profiling in response to heterofermentative LAB additives.

In this study, LAB‐inoculated groups, particularly LH and LBLH, exhibited lower DM content after 60 days of ensiling compared with the CK group, likely attributable to increased acid production during fermentation. However, the DM loss remained within the acceptable limit of 6% recommended for silage preservation (Villa et al. 2020), indicating effective preservation. The lower WSC content observed in LAB‐inoculated groups compared with the CK group reflects accelerated fermentation, in which WSC is rapidly converted into LA or AA during the early ensiling phase. Proteolysis, the enzymatic breakdown of protein, predominantly occurs in early fermentation, affecting CP content (Jin, Tahir, et al. 2024). Notably, CP content declined more sharply in the LH and LBLH groups, suggesting that heterofermentative LAB alters microbial community dynamics and nitrogen metabolism within the silage. Ammonia‐N is a key indicator of protein degradation mostly arising from plant proteases and *Clostridium* activity (Jin, Wang, and Li 2024). Ammonia‐N was significantly lower in the LH and LBLH groups than in the CK and LB groups. Although CP declined more in LH and LBLH, this pattern most likely reflects a dilution effect driven by better preservation of non‐nitrogen constituents in these groups, rather than a loss of nitrogen (Xu et al. 2023), as evidenced by the significantly lower ammonia‐N. Consistent with this interpretation, the reduction in ammonia‐N indicates that a larger share of total N remained as true protein and peptide‐N instead of being deaminated to ammonia, so while CP% decreased on a DM basis, protein preservation actually improved. From a nutritional standpoint, the modest decline in CP% is unlikely to impair dietary protein supply, because the form of N (lower ammonia‐N, greater true protein/peptide‐N) is more favourable for rumen capture and downstream metabolisable protein (Luasiri et al. 2024), and any small percentage‐point change can be readily balanced at the ration level, whereas the improved aerobic stability delivered by LBLH is expected to enhance intake and reduce nutrient losses during feed‐out. ADF content decreased in the LB group, but increased in the LBLH group compared to other groups. *Lactobacillus buchneri* , a key heterofermentative LAB, can degrade ADF by producing lignocellulolytic enzymes that break down cell wall carbohydrates into WSC (Wang et al. 2023). However, the higher ADF in the LBLH group may reflect a shift in microbial metabolism, where the combined action of these strains alters fibre degradation patterns. For instance, LH may modulate the enzymatic activity of LB, leading to preferential utilisation of other carbohydrate fractions (e.g., hemicellulose or WSC) over ADF, thereby preserving fibre content. Alternatively, the co‐inoculation may enhance microbial efficiency, redirecting metabolic pathways toward the production of fermentation end products (e.g., AA) rather than extensive fibre breakdown. LAB‐inoculated groups exhibited higher AA content and lower BA content than the CK group after 60 days of ensiling. Heterofermentative LAB are known to metabolise LA into AA, 1,2‐propanediol, carbon dioxide and ethanol under anaerobic conditions (Li et al. 2021). Since BA is a harmful fermentation byproduct produced by undesirable bacteria, its reduction in LAB‐inoculated groups indicates suppressed undesirable microbial activity and improved fermentation quality. Notably, LA and PA contents did not differ significantly among groups. This may be explained by the fact that sweet sorghum contains abundant WSC, supporting rapid LA production even without inoculation (Usman et al. 2025). Hence, the natural epiphytic LAB community likely produced sufficient LA to reach levels comparable to the inoculated groups. Similarly, PA production is often associated with specific bacterial populations such as *Propionibacterium*, which may not have been strongly stimulated by the inoculants used, leading to comparable concentrations across treatments.

When a silo is opened or silage is exposed to air, oxidation of fermentation acids and other compounds occurs due to the activity of yeasts, aerobic bacteria and moulds (Wilkinson and Davies 2013). This aerobic deterioration leads to substantial nutrient losses and reduced feed quality. Enhancing aerobic stability of silages is therefore critical to minimise spoilage and ensuring safe and toxins‐free silage for ruminants. In this study, the LBLH group resulted in significantly higher aerobic stability hours along with a longer time to reach the maximum temperature compared to other groups. Interestingly, the LBLH group delayed the nutritional and fermentation loss by sustaining lower fibre contents, pH value, yeast counts and higher LA, AA and WSC content compared to other groups at 22 days of aerobic exposure. The superior performance of co‐inoculation with LB and LH beyond the additive effects of single strains likely arises from metabolic complementarities between the two species. *Lactobacillus buchneri* is well known for its ability to convert LA into AA and 1,2‐propanediol, thereby enhancing aerobic stability by inhibiting spoilage yeasts and moulds (Kleinschmit and Kung Jr 2006), whereas *Lactobacillus hilgardii* contributes by metabolising residual sugars and 1,2‐propanediol into further metabolites such as PA, which may provide additional antifungal properties (Wang et al. 2020). Together, this cross‐feeding interaction not only broadens the spectrum of inhibitory metabolites but also prevents the accumulation of intermediates, improving fermentation efficiency and reducing the risk of undesirable microbial growth (Nair et al. 2020). This complementary division of metabolic labour likely underpins the synergistic effects observed in LBLH, leading to improved aerobic stability, detoxification potential and overall silage safety compared to single‐strain inoculations. Similar to these results, da Silva et al. (2021) reported that the combined inoculation of *Lactobacillus hilgardii* 4785 and *Lactobacillus buchneri* 40,788 enhanced the aerobic stability of corn silage with higher AA production than silages treated with either strain alone or left untreated (da Silva et al. 2021). WSC serves as a readily available substrate for spoilage microbes during aerobic exposure, driving increases in temperature and pH (Addah et al. 2012). Consistent with this, a progressive decline in WSC content was observed across all groups with prolonged aerobic exposure, reflecting its consumption by spoilage microbes. Notably, this depletion was attenuated in the LBLH group, which corresponded with its reduced populations of cultivable yeasts and aerobic bacteria, alongside an elevated count of LAB, relative to the other groups. This implies a more effective suppression of spoilage microbes and enhanced preservation of fermentable substrates in the LBLH group.

The decrease in fungal diversity and increase in bacterial diversity observed in LAB‐inoculated groups (particularly in LH and LBLH) during prolonged aerobic exposure, compared to the CK group, aligns with previous findings (Tahir et al. 2025). A possible explanation is that LAB combinations thrive in acidic environments because they produce beneficial organic acids and exhibit antibacterial properties. This helps suppress pathogenic bacteria, allowing beneficial bacteria to dominate and improve silage quality (Jin, Tahir, et al. 2024; Wang et al. 2022). These results are consistent with microbial count data: in LAB‐inoculated groups – especially LBLH – LAB population increased more rapidly, while aerobic bacteria and yeast counts declined faster after 60 days of ensiling. Conversely, LAB counts decreased more slowly, and the rise in aerobic bacteria and yeasts was also delayed during aerobic exposure. This suggests a synergistic effect of the LBLH combination in enhancing native LAB growth and delaying spoilage. Apart from this, *Pseudomonas psychrotolerans* was most abundant in the LB group, whereas *Gluconobacter oxydans* and *Lactobacillus buchneri* were predominant in the LH and LBLH groups. *Gluconobacter oxydans* and *Lactobacillus buchneri* are known to promote the production of AA (da Silva et al. 2022; Muck et al. 2018), which explains why the aerobic stability of the LBLH group was higher than that of other groups. In the CK group, *Saccharomyces* and *Kazachstania* were the dominant fungal genera, whereas LAB‐inoculated groups were characterised by *Zygosaccharomyces* and *Penicillium*. After 8 days of aerobic exposure, *Saccharomyces cerevisiae* and *Kazachstania humilis* were the most prevalent species in the CK group, but by day 22, they were replaced by *Zygosaccharomyces bisporus*. *Zygosaccharomyces* and *Kazachstania* species are among the most problematic food spoilage yeasts (Faherty et al. 2019; Galeote et al. 2013). *Zygosaccharomyces bisporus* is known for its ability to tolerate high osmotic stress, and it can survive and grow in environments with high sugar concentrations (Sharma and Sharma 2017), whereas *Kazachstania humilis* is generally recognised as a spoilage species (Santos et al. 2017; Tahir et al. 2025). In LAB‐inoculated groups, *Fusarium equiseti* (in the LB group) and *Zygosaccharomyces bailii* (in the LH and LBLH groups) were the dominant fungal species at 8 days of aerobic exposure. By day 22, *Zygosaccharomyces bisporus* became prevalent in the LB group, while *Zygosaccharomyces bailii* and *Penicillium paneum* became dominant in the LH and LBLH groups. The observed fungal succession patterns in LAB‐inoculated silages, characterised by the early dominance of *Zygosaccharomyces bailii*, especially in LH and LBLH groups at day 8 and subsequent shifts to *Penicillium paneum* by day 22, can be attributed to their distinct physiological adaptations and interactions with fermentation metabolites. *Zygosaccharomyces bailii* thrived initially due to its exceptional acid tolerance enabled by proton efflux pumps and ability to utilise LA as a carbon source, allowing it to dominate the acidic environment created by LAB inoculation (Stratford et al. 2013). Its osmotolerant nature further facilitated growth on residual sugars in the silage, as evidenced by the reduction in WSC content during aerobic exposure. However, as *Zygosaccharomyces bailii* metabolised LA, the resulting pH increase created favourable conditions for *Penicillium paneum*, which exhibits moderate acid tolerance and xerotolerance that becomes advantageous as silage moisture declines (O'Brien et al. 2006). These findings highlight how spoilage fungal succession in silage is governed by an interplay between microbial physiological traits, fermentation acid profiles and environmental parameters, emphasising the need for tailored inoculation strategies that address both initial acidification and long‐term aerobic stability.

To elucidate the nutritional and fermentation losses in sweet sorghum silage after aerobic exposure, predicting functional profiles is essential for understanding the impact of LAB additives on silage quality (Zhao et al. 2022). At the first‐level pathway, metabolism was higher in all groups, while second‐level pathways, including xenobiotics biodegradation and metabolism, carbohydrate metabolism, terpenoid and polyketide metabolism, and amino acid metabolism, were significantly enhanced in silages after aerobic exposure. The metabolic functions of heterofermentative LAB were primarily assessed based on carbohydrate metabolism and fermentation products. Following aerobic exposure, all pathways were down‐regulated in the CK and LH groups, whereas the LB group displayed broad up‐regulation of nearly all pathways at 8 days, with the exception of Ko00363. This up‐regulation may reflect the metabolic versatility of *Lactobacillus buchneri* , which is known to activate alternative carbohydrate and secondary metabolic pathways under aerobic stress. The enhanced expression of xenobiotic biodegradation and carbohydrate metabolism pathways in the LB group suggests that *Lactobacillus buchneri* may respond to oxygen ingress by redirecting metabolism toward the utilisation of residual substrates and the detoxification of inhibitory compounds. The Ko00363 pathway, which facilitates the degradation of toxic compounds into less harmful products, was uniquely enriched in the combined LBLH group. The enrichment of the Ko00363 pathway in the combined LBLH group suggests potential practical implications for silage safety beyond just improving aerobic stability. Since this pathway enables microbial detoxification of bisphenols and related endocrine‐disrupting compounds (Saffari Ghandehari et al. 2023), its presence in silage could help reduce contamination risks from environmental pollutants that may enter forage crops through soil, water, or plastic storage materials. Given that bisphenols can persist in agricultural systems and potentially transfer into livestock feed (Rapiya et al. 2025), their microbial degradation during ensiling may contribute to safer animal feed by lowering residual toxin levels. While further validation is needed to confirm the extent of bisphenol breakdown in silage, this mechanism could be particularly valuable in regions with high industrial pollution or where plastic‐wrapped silage is common. Thus, the combined LB and LH inoculation may offer a dual benefit – enhancing silage preservation while also mitigating hidden chemical risks in feed safety. Overall, this study demonstrated that co‐inoculation with LB and LH enhances the aerobic stability of sweet sorghum silage. This effect is attributed to reduced yeast populations and increased AA production, driven by the high abundance of *Gluconobacter oxydans* and *Lactobacillus buchneri* during aerobic exposure. As a result, the dominance of specific yeasts and opportunistic facultative anaerobic bacteria was effectively suppressed.

## 5 Conclusion

This study confirms that heterofermentative LAB inoculation increased AA content, while reducing the pH value and BA and ammonia‐N levels. Among the tested groups, the LBLH group demonstrated the highest aerobic stability (462 h). During aerobic exposure, the LBLH group delayed the nutritional and fermentation loss by maintaining lower pH and ammonia‐N levels while preserving higher LA and AA contents compared to other groups. Microbial analysis revealed that *Gluconobacter oxydans* , *Zygosaccharomyces bailii* and *Penicillium paneum* were the dominant species in the LBLH group. These findings suggest that the LBLH inoculant not only improves fermentation quality but also enhances aerobic stability, thereby reducing nutrient losses during the aerobic exposure of sweet sorghum silage.

## Author Contributions

**Muhammad Tahir:** designing, methodology, formal analysis, writing – original draft. **Tianwei Wang:** formal analysis, writing – review and editing, funding acquisition. **Zhiquan Liu** and **Yongkai Luo:** resources. **Zhihui Fu** and **Shanji Liu:** data curation and visualisation. **Jin Zhong:** project administration, funding acquisition.

## Conflicts of Interest

The authors declare no conflicts of interest.

## Supporting information


**Figure S1:** Microbial counts of sweet sorghum silage after 60 days of ensiling in response to heterofermentative lactic acid bacteria additive. (A) lactic acid bacteria, (B) aerobic bacteria, (C) yeasts. CK, sterilised water; LB, *Lactobacillus buchneri* NX205; LH, *Lactobacillus hilgardii* M1814; LBLH, combination of LB and LH. Data is the mean of three replicates. The small letters indicate the statistical difference among treatments which was employed at 0.05 probability level.

**Figure S2:** Microbial counts of sweet sorghum silage after aerobic exposure in response to heterofermentative lactic acid bacteria additive. (A) lactic acid bacteria, (B) aerobic bacteria, (C) yeasts. CK, sterilised water; LB, *Lactobacillus buchneri* NX205; LH, *Lactobacillus hilgardii* M1814; LBLH, combination of LB and LH; T, treatment; D, aerobic exposure days; T × D; interactive effect between treatments and aerobic exposure days. Data is the mean of three replicates. The small letters indicate the statistical difference among treatments at same time point, which was employed at 0.05 probability level.

**Table S1:** Alpha diversity of bacterial community of sweet sorghum silage after aerobic exposure.
**Table S2:** Alpha diversity of fungal community of sweet sorghum silage after aerobic exposure.

## Data Availability

The raw sequencing files and associated metadata have been deposited in the National Center for Biotechnology Information (NCBI) Sequence Read Archives (bacterial community, PRJNA1266375; fungal community, PRJNA1266384).
