# Supplementary figures and images for: Heterofermentative Lactic Acid Bacteria Enhance the Aerobic Stability of Sweet Sorghum Silage
Source: Microb Biotechnol. 2025 Nov 8;18(11):e70262. doi: 10.1111/1751-7915.70262 (PMC12595604; doi:10.1111/1751-7915.70262)

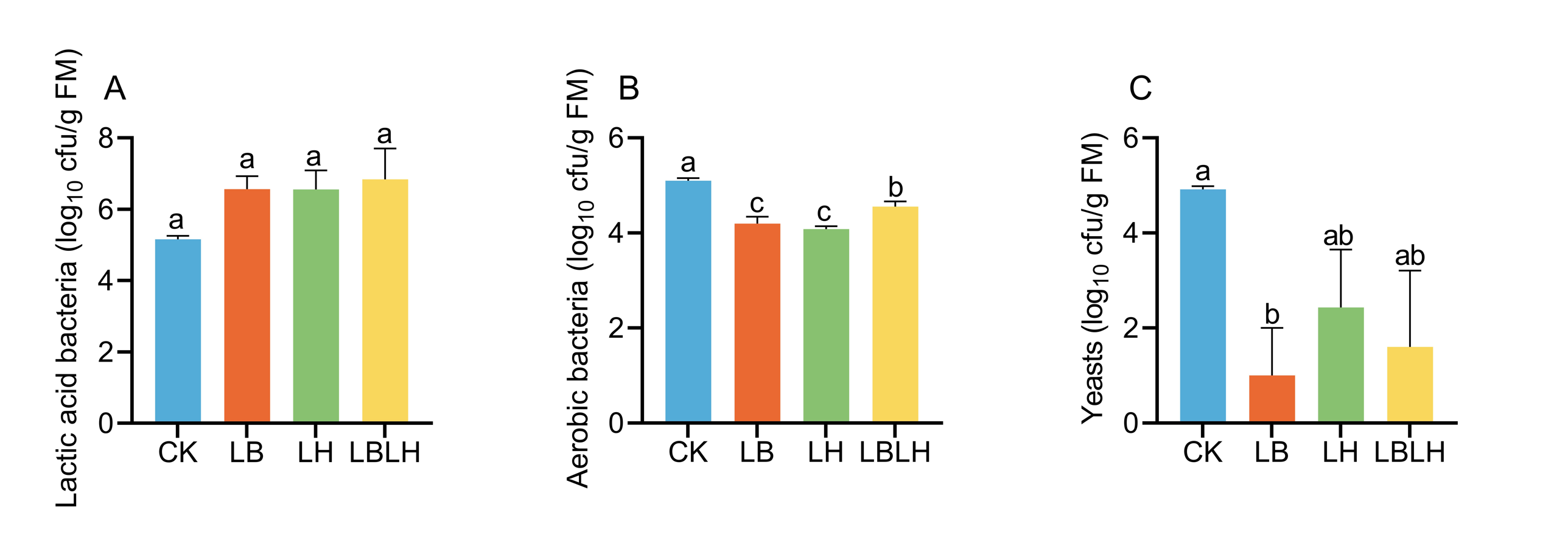

Supplement: Supplementary file 1 — Figure S1: Microbial counts of sweet sorghum silage after 60 days of ensiling in response to heterofermentative lactic acid bacteria additive. (A) lactic acid bacteria, (B) aerobic bacteria, (C) yeasts. CK, sterilised water; LB, Lactobacillus buchneri NX205; LH, Lactobacillus hilgardii M1814; LBLH, combination of LB and LH. Data is the mean of three replicates. The small letters indicate the statistical difference among treatments which was employed at 0.05 probability level. [file MBT2-18-e70262-s001.jpg]

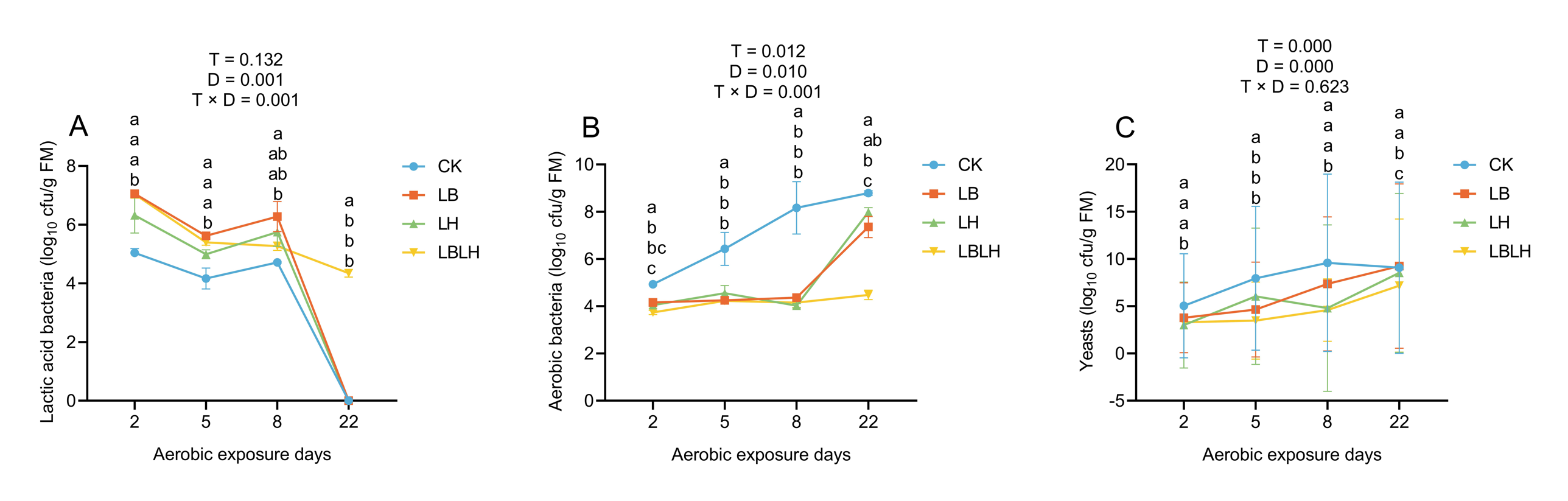

Supplement: Supplementary file 2 — Figure S2: Microbial counts of sweet sorghum silage after aerobic exposure in response to heterofermentative lactic acid bacteria additive. (A) lactic acid bacteria, (B) aerobic bacteria, (C) yeasts. CK, sterilised water; LB, Lactobacillus buchneri NX205; LH, Lactobacillus hilgardii M1814; LBLH, combination of LB and LH; T, treatment; D, aerobic exposure days; T × D; interactive effect between treatments and aerobic exposure days. Data is the mean of three replicates. The small letters indicate the statistical difference among treatments at same time point, which was employed at 0.05 probability level. [file MBT2-18-e70262-s002.jpg]
