# Supplementary material for: Heterofermentative Lactic Acid Bacteria Enhance the Aerobic Stability of Sweet Sorghum Silage
Source: Microb Biotechnol. 2025 Nov 8;18(11):e70262. doi: 10.1111/1751-7915.70262 (PMC12595604; doi:10.1111/1751-7915.70262)
Supplement: Supplementary file 3 — Table S1: Alpha diversity of bacterial community of sweet sorghum silage after aerobic exposure. Table S2: Alpha diversity of fungal community of sweet sorghum silage after aerobic exposure. [file MBT2-18-e70262-s003.docx]

| Table S1. Alpha diversity of bacterial community of sweet sorghum silage after aerobic exposure. | | | | | | | |
| --- | --- | --- | --- | --- | --- | --- | --- |
| Items | Treatment | Aerobic exposure days | | SEM | Significance | | |
|  |  | D8 | D22 |  | T | D | T × D |
| Observed | CK | 314.66^abA^ | 332.00^aA^ | 16.699 | 0.258 | 0.81 | 0.321 |
|  | LB | 424.66^aA^ | 304.67^aB^ |  |  |  |  |
|  | LH | 257.33^abA^ | 272.00^aA^ |  |  |  |  |
|  | LBLH | 215.00^bB^ | 263.67^aA^ |  |  |  |  |
| Chao1 | CK | 373.32^aA^ | 365.30^aA^ | 19.597 | 0.134 | 0.728 | 0.586 |
|  | LB | 426.19^aA^ | 324.77^aB^ |  |  |  |  |
|  | LH | 276.02^aA^ | 288.26^aA^ |  |  |  |  |
|  | LBLH | 218.37^aB^ | 266.75^aA^ |  |  |  |  |
| ACE | CK | 373.90^aA^ | 365.92^aA^ | 19.608 | 0.137 | 0.731 | 0.58 |
|  | LB | 427.48^aA^ | 325.29^aB^ |  |  |  |  |
|  | LH | 276.29^aA^ | 288.93^aA^ |  |  |  |  |
|  | LBLH | 219.19^aB^ | 268.05^aA^ |  |  |  |  |
| Shannon | CK | 2.8558^abA^ | 2.65^aA^ | 0.098 | 0.146 | 0.362 | 0.103 |
|  | LB | 3.57^aA^ | 2.42^aB^ |  |  |  |  |
|  | LH | 1.99^bA^ | 1.72^bA^ |  |  |  |  |
|  | LBLH | 1.67^bA^ | 1.99^bA^ |  |  |  |  |
| Simpson | CK | 0.16^aA^ | 0.11^bA^ | 0.026 | 0.086 | 0.308 | 0.505 |
|  | LB | 0.09^aB^ | 0.16^bA^ |  |  |  |  |
|  | LH | 0.22^aB^ | 0.40^aA^ |  |  |  |  |
|  | LBLH | 0.34^aA^ | 0.37^aA^ |  |  |  |  |
| Coverage | CK | 1.00^aA^ | 1.00^aA^ | 0.000 | 0.596 | 1.000 | 0.461 |
|  | LB | 1.00^aA^ | 1.00^aA^ |  |  |  |  |
|  | LH | 1.00^aA^ | 1.00^aA^ |  |  |  |  |
|  | LBLH | 1.00^aA^ | 1.00^aA^ |  |  |  |  |
| CK, sterilized water; LB, *Lactobacillus buchneri* NX205; LH, *Lactobacillus hilgardii* M1814; LBLH, combination of LB and LH; T, treatment; D, aerobic exposure days; T × D; interactive effect between treatments and aerobic exposure days; SEM, standard error mean. Small letter represents the significance within same column among treatments, whereas capital letters indicate the significance within same row among aerobic exposure days. The significance was employed as *P* < 0.05. | | | | | | | |

| Table S2. Alpha diversity of fungal community of sweet sorghum silage after aerobic exposure. | | | | | | | |
| --- | --- | --- | --- | --- | --- | --- | --- |
| Items | Treatment | Air exposure days | | SEM | Significance | | |
|  |  | D8 | D22 |  | T | D | T× D |
| Observed | CK | 22.00^bA^ | 76.33^aA^ | 8.37 | 0.394 | 0.559 | 0.004 |
|  | LB | 215.66^aA^ | 64.00^aA^ |  |  |  |  |
|  | LH | 31.33^bA^ | 18.33^aA^ |  |  |  |  |
|  | LBLH | 43.33^bA^ | 39.33^aA^ |  |  |  |  |
| Chao1 | CK | 22.00^bA^ | 79.00^aA^ | 8.838 | 0.411 | 0.556 | 0.005 |
|  | LB | 215.66^aA^ | 64.00^aA^ |  |  |  |  |
|  | LH | 34.66^bA^ | 18.33^aA^ |  |  |  |  |
|  | LBLH | 47.66^bA^ | 42.66^aA^ |  |  |  |  |
| ACE | CK | 22.00^bA^ | 79.00^aA^ | 8.838 | 0.411 | 0.556 | 0.005 |
|  | LB | 215.66^aA^ | 64.00^aA^ |  |  |  |  |
|  | LH | 34.66^bA^ | 18.33^aA^ |  |  |  |  |
|  | LBLH | 47.66^bA^ | 42.66^aA^ |  |  |  |  |
| Shannon | CK | 1.12^bA^ | 1.72^aA^ | 0.177 | 0.549 | 0.717 | 0.16 |
|  | LB | 2.80^aA^ | 1.17^aA^ |  |  |  |  |
|  | LH | 0.92^bA^ | 0.81^aA^ |  |  |  |  |
|  | LBLH | 1.16^bA^ | 1.50^aA^ |  |  |  |  |
| Simpson | CK | 0.47^aA^ | 1.72^aA^ | 0.055 | 0.890 | 0.511 | 0.209 |
|  | LB | 0.13^bA^ | 1.17^aA^ |  |  |  |  |
|  | LH | 0.51^aA^ | 0.81^aA^ |  |  |  |  |
|  | LBLH | 0.38^abA^ | 1.50^aA^ |  |  |  |  |
| Coverage | CK | 1.00^aA^ | 1.00^aA^ | 0.000 | 0.000 | 0.000 | 0.000 |
|  | LB | 1.00^aA^ | 1.00^aA^ |  |  |  |  |
|  | LH | 1.00^aA^ | 1.00^aA^ |  |  |  |  |
|  | LBLH | 1.00^aA^ | 1.00^aA^ |  |  |  |  |
| CK, sterilized water; LB, *Lactobacillus buchneri* NX205; LH, *Lactobacillus hilgardii* M1814; LBLH, combination of LB and LH; T, treatment; D, aerobic exposure days; T × D; interactive effect between treatments and aerobic exposure days; SEM, standard error mean. Small letter represents the significance within same column among treatments, whereas capital letters indicate the significance within same row among aerobic exposure days. The significance was employed as *P* < 0.05. | | | | | | | |
